# Diverging transposon activity among polar bear sub-populations inhabiting different climate zones

**DOI:** 10.1186/s13100-025-00387-4

**Published:** 2025-12-12

**Authors:** Alice M. Godden, Benjamin T. Rix, Simone Immler

**Affiliations:** School of Biological Sciences, University of East Anglia, Norwich Research Park, Norwich, NR4 7TJ UK

**Keywords:** Transposable elements, Thermal stress, Adaptive response, Ursus, Polar bear

## Abstract

A new subpopulation of polar bears (*Ursus maritimus*) was recently discovered in the South-East of Greenland. This isolated colony inhabits a warmer climate zone, akin to the predicted future environments of polar bears with vastly reduced sea ice habitats, rendering this population of bears particularly important. Over two-thirds of polar bears will be extinct by 2050 with total extinction predicted by the end of this century, therefore understanding possible mechanisms of adaptation via genomic analyses and preservation are critical. Transposable elements (TEs) are mobile elements that may play a role in an adaptive response to environmental challenges. We analysed transcriptome data from polar bear sub-populations in cooler North-East (NEG) and warmer South-East Greenland (SEG) to compare TE activity between the two populations and its correlation with temperature and associated changes in gene expression. We identified activity hotspots in the genome of regions with significantly differentially expressed TEs. LINE family TEs were the most abundant, and most differentially expressed and divergent in the SEG population compared to reference TEs. We report a significant shift in TE activity and age, with younger more abundant TEs in the SEG populations. Differentially expressed genes in SEG populations were linked to *Foxo* signalling, ageing and metabolic pathways. Our results provide insights into how a genomic response at the TE level may allow the SEG subpopulations to adapt and survive to climate change and provides a useful resource for conservation in polar bears.

**Supplementary Information:**

The online version contains supplementary material available at 10.1186/s13100-025-00387-4.

## Background

Global average temperatures in 2024 were more than 1 °C above the pre-industrial era [1]. The Arctic Ocean current is at its warmest in the last 125,000 years, and temperatures continue to rise [2, 3]. In the South East of Greenland (SEG), the ice sheet margin is rapidly receding, causing vast ice and habitat loss [4]. North-East Greenland (NEG) is an Arctic tundra, whereas SEG is covered by forest tundra [5]. The SEG climate has high levels of precipitation, wind, and steep coastal mountains [6]. This challenging habitat has led to severe warnings for the survival of the polar bear *Ursus maritimus*, with a 71% probability of a reduction in population size by over 90% within 40 years. The International Union for Conservation of Nature (IUCN) has listed the polar bear as a vulnerable species [7], and understanding possible ways of adaptation to climate change in such an enigmatic animal at the genome level is key.

Environmental stress is reported to have a significant impact on genome structure, including the expression and mobilisation of transposable elements (TEs) [8]. TEs are mobile genetic elements that can shape evolution through self-replication and insertion events in the genome, which in turn may change gene expression patterns [9, 10]. TEs inserted into or near a promoter region may alter the expression of genes and generate novel regulatory networks [11]. TEs make up 38.1% of the genome in polar bears [12], which is similar to the giant panda *Ailuropoda melanoleuca* at 37%, and 36.1% of the dog *Canis familiaris* genome [13], and hence TEs may play an important part in the process of adaptation. Current data show that over 150,000 genome structural variants have been generated by TEs, driving variation in *ursine* and *tremarctine* bears [12]. The aim of this study was to test if TE divergence between SEG and NEG polar bear populations may explain genetic differences and variation in gene expression between these sub-populations using a published transcriptome dataset [14]. We included information on temperature variation into our analyses to test for a possible association between temperature and TE activity.

## Methods

###  Meteorological analysis

We collected and analysed meteorological data on the climate in NEG and SEG from the Danish Meteorological Institute [15]. SEG locations included: Aputiteeq, Tasiilaq, Mittarfik, Kulusuk, Ikermiit and Qaqortoq. NEG locations included: Ittoqqortoormiit, Daneborg, Port of Denmark and Station Nord (see Suppl. File 1 for latitude and longitude co-ordinates). Temperature data covering the highest, and lowest recorded temperatures each year from 1958 to 2024 were included and the data was plotted with Tableau Public version 2024.1.

### RNA-seq data

Transcriptome data from blood samples of adult polar bears were downloaded from the European Nucleotide Archive under accession no. PRJNA669153 [14] (see Suppl. File 2 for sample accession numbers and metadata) and aligned to the published reference polar bear genome ASM1731132v1. As described in the original publication [14], RNA-sequencing (RNA-seq) was based on total RNA and haemoglobin depletion step by CRISPR-Cas9 was used during library preparation. The libraries were sequenced on Illumina HiSeqX to generate 151 bp paired end reads [14]. Our downstream analyses included data from 17 adult samples, comprised of five female and seven male NEG bears, and two male and three male SEG bears. From these data, an average of 71 million reads per sample were successfully mapped to the genome.

NF-core RNA-Seq pipeline version 3.9 was used to analyse the RNA-seq data (https://nf-co.re/rnaseq/1.4.2/) [16]. Genome and annotation files were used and accessed as follows: ASM1731132v1 (GCF_017311325.1) GCF_017311325.1_ASM1731132v1_genomic.fna, and annotation GCF_017311325.1_ASM1731132v1_genomic.gtf https://www.ncbi.nlm.nih.gov/datasets/genome/GCF_017311325.1/). ASM1731132v1 is the most recent genome assembly and was used here as the reference genome. All differential expression analyses were comparing the intercept of NEG versus SEG population, to observe impact on the Southern population of bears.

### Differential TE expression analysis

To identify locus-specific expression of TEs and differentially expressed (DE) TE species, we used *Telescope* v1.0.3, a software previously tested on blood sample transcriptome data to profile differential TE expression at the species and single locus level [17]. Telescope was used as follows: telescope assign input.bam \ ASM1731132v1_rmsk_TE.gtf \ --attribute transcript_id \ --outdir telescope. To produce the TE gtf annotation file for Telescope pipeline, the Perl scripts from TETranscripts [18] were used as follows: *perl makeTEgtf.pl -c 1 -s 2 -e 3 -o 6 -t 4 ASM1731132v1_rmsk >ASM1731132v1_rmsk_TE.gtf.* The file ASM1731132v1_rmsk was a bed file downloaded from the UCSC table browser from here: https://genome-euro.ucsc.edu/cgi-bin/hgTables with *Clade: Polar bear (UCSC 2021)*,* Genome: ASM1731132v1 Mar. 2021*,* Assembly: polar bear (UCSC 2021)*,* Group: Variation and Repeats*,* Track: Repeatmasker*,* Region: Genome*, and downloaded output as a bed file format. The raw counts of TEs from Telescope and differential expression analysis from DESeq2 can be found in Suppl. files 3 and 4 respectively. The results modelled with temperature effect as a covariate in the model on TE differential expression in DESeq2 analysis are contained in Suppl. File 5.

###  Repeat landscape analysis of TEs

In addition to differential expression of TEs, we used *RepeatMasker v*4.1.1 [19] and *RepeatModeler* v2.0.3 [20] to generate repeat landscapes. The first step in this process was to generate de novo transcriptome assemblies from the raw RNA-seq fastq files. Raw RNA-seq reads were trimmed with Fastp v0.23.2 [21] with default settings for paired end reads input. To generate the *de novo* assemblies with our RNA-seq data we used the Spades v4.0.0[22] pipeline as follows: spades.py \ --rna \ −1 ${base}_1.trimmed.fq.gz \ −2 ${base}_2.trimmed.fq.gz \ -o assemblies/${base} \ -t 5 \ -m 50. The fasta file generated from each sample was then processed with RepeatMasker.

The default setting in RepeatMasker for species − 2903 (*Ursus maritimus*) is using the UrsMar_1.0 reference assembly. We replaced this by the latest genome assembly available, namely ASM1731132v1. To generate a custom library of transposable elements (TEs) for the polar bear genome, we used RepeatModeler and the GCF_017311325.1_ASM1731132v1_genomic.fna assembly as a reference. First, we prepared the reference genome using the BuildDatabase utility: BuildDatabase -name custom_repeats_db_ASM1731132v1 -engine ncbi GCF_017311325.1_ASM1731132v1_genomic.fna. Following database creation, we ran RepeatModeler with 40 parallel threads to identify and assemble the TEs: RepeatModeler -database custom_repeats_db_ASM1731132v1 -pa 40 -engine ncbi. The reference database generated can be found here: https://zenodo.org/records/17573136 file “custom_repeats_db_ASM1731132v1-families.fa”. The RepeatMasker pipeline was then run as follows: RepeatMasker input.assembly.fasta \ -lib custom_repeats_db_ASM1731132v1-families.fa\ -dir outputdirectory \ -pa 5 \ -s \ -a \ -nolow \ -no_is \. To profile Kimura distances and assess evolutionary divergence of nucleotide substitutions [23], aligned outputs (.align) from RepeatMasker were analysed with calcDivergenceFromAlign.pl as follows: calcDivergenceFromAlign.pl -s genome.fa.align >genome_divsum.

To test for a significant shift in Kimura substitution values for TEs, R scripts for general linear mixed modelling were used with R packages: *MASS* v7.3–60.0.0.1 [24] *pscl* v1.5.9 [25], with a model assuming negative binomial error distribution (Count ~ Condition * Div + (1|Sample) where *Count* is the TE abundance at the Kimura substitution level, *Condition* is enrichment in SEG population and *Div* are the Kimura substitution levels) showing the best fit due to overdispersion in the TE count data (see Suppl. Table 1 for model comparisons).

### Differential gene expression analysis

To profile differential expression of genes, the nextflow pipeline nf-core/rnaseq version 3.9 pipeline was used. This was executed as follows: nextflow run nf-core/rnaseq -r 3.9 --input samplesheet.csv --outdir rnaseq -c bear.conf -profile singularity. We used the bam files generated with STAR aligner and fed these into Salmon for downstream quantification of raw counts data. STAR generated BAM files with the following parameters: ID: STAR PN: STAR VN:2.7.10a CL: STAR --runThreadN 12 --runRNGseed 0 --genomeDir star --readFilesIn SAMN16454141_1_val_1.fq.gz SAMN16454141_2_val_2.fq.gz --readFilesCommand zcat --outFileNamePrefix SAMN16454141. --outSAMtype BAM Unsorted --outSAMstrandField intronMotif --outSAMattributes NH HI AS NM MD --outSAMattrRGline ID: SAMN16454141 SM: SAMN16454141 --outFilterMultimapNmax 20 --alignSJDBoverhangMin 1 --sjdbGTFfile filtered_with_gene_id.gtf --quantMode TranscriptomeSAM --quantTranscriptomeBan Singleend --twopassMode Basic (raw count data in Suppl. File 6, DESeq2 data in Suppl. File. 7-for population effect, Suppl. File. 8- for temperature effect).

### Differential TE expression analysis

We used *DESeq2* [26] in *RStudio* v1.4.1717 with R v4.1.1 [27] to run a differential expression analyses between SEG and NEG bears. To account for spurious reads, any gene or TE alignment with less than ten reads was discarded before running *DESeq2*. The input data is organised into a *DESeqDataSet* object (dds) containing the raw count matrix of expressed TEs and genes together with the associated sample metadata and experimental design formula. The baseline model used to generate the dds object was ~ sex + population, where “population” refers to the NEG or SEG group. This design accounts for potential sex-specific differences in expression while testing for population effects. To investigate the influence of temperature on TE expression, we extended the model to include annual mean temperature (average temperature in 2016, the year prior to sampling in 2017, measured at the town nearest to each bear’s location). The resulting design was ~ sex + temperature + population. Results were extracted as follows: *res_temperature <- results(dds*,* name="temperature”)*. As location and temperature were colinear, the additive model was used without an interaction term to avoid redundancy. The significance threshold for DE genes and TEs was log_2_ fold change > 1 and a *p*_adj_ < 0.05. For GO terms and KEGG pathway analyses, we used *ShinyGO* v0.77 [28] on significantly DE genes with a background list of all genes found to be expressed in the data, and figures were generated using custom Python scripts.

### PCA analysis

We used the dds object to perform a Principal Component Analysis (PCA) using rlog transformation and with sex, population and latitude as factors and generated cluster figures. To analyse the clustering of our samples independently of any annotation or alignment, raw RNA-seq reads were mapped to 10-kb bins across the whole genome. To do this we used Deeptools [29] v3.5.2 with the MultiBamSummary and PCA plotting features with --binSize 10000 for 10-kb binning.

### Software and analysis

All R scripts and Python scripts used to generate figures can be found at: https://github.com/alicegodden/polarbear/. The following packages were used in the analysis and presentation of the data: *DESeq2* 1.34.1 [26], *Tidyverse* 2.0.0 [30], *EnhancedVolcano* 1.12.0 [31], *ggplot2* 3.4.2 [32], *Readr* 2.1.4 [33], *tidyr* v1.3.0 [34], *viridis* v0.6.4 [35], *reshape* [36], *hrbrthemes*v0.8.7[37], and *gridExtra*v2.3[38],, *MASS* v7.3–60.0.0.1 [24] *pscl* v1.5.9 [25].

### TE gene loci intersection

To analyse the overlap of significantly DE TEs with genomic features (coding sequence CDS, exon, gene, lncRNA, mRNA, pseudogene, snRNA and transcript), we compared the Telescope results with gtf and bed file annotations using bedtools intersect [39]. Significantly enriched DE genes overlapping with DE TEs in the SEG population were used for GO term analysis.

## Results

### Warmer temperatures in Southeast compared to Northeast Greenland

**Fig. 1:**
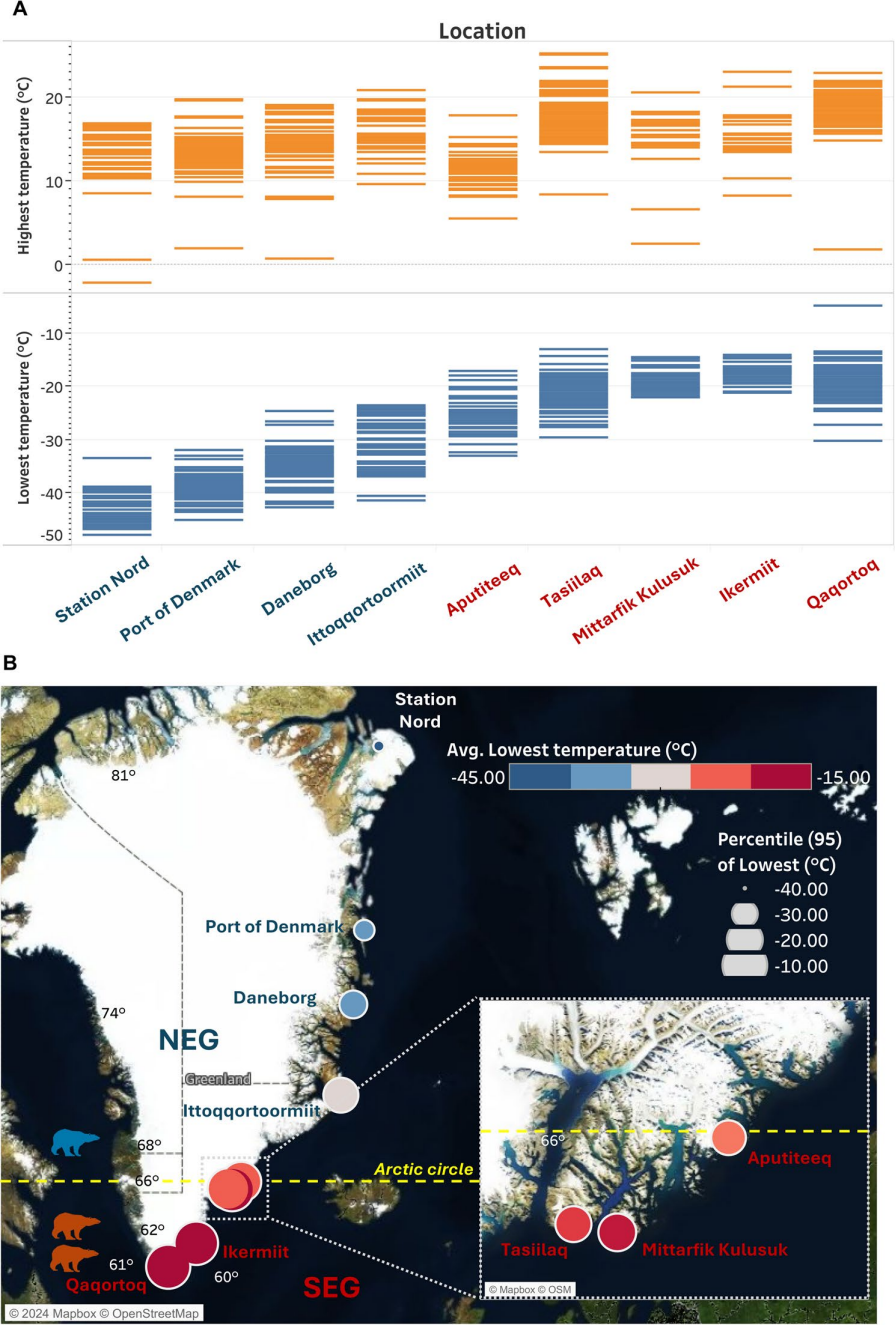
Meteorological observation data of temperature from the DMI. **A** Highest and lowest observed temperatures from 1958–2024, listed from Northernmost to most Southern. **B** Colour of points indicates the average lowest observed temperature, with the size of point showing the variance at the 95th percentile. SEG locations included: Aputiteeq, Tasiilaq, Mittarfik, Kulusuk, Ikermit and Qaqortoq. NEG locations included: Ittoqqortoormiit, Daneborg, Port of Denmark and Station Nord. In this study north of 64^o^ was considered NEG. The bear icons are indicative of the latitude where they were sampled, red meaning SEG and blue NEG. The Arctic circle is denoted by a dashed yellow line at 66^o^

The meteorological observation data from Danish Meteorological Institute (DMI) revealed substantial variation in average lowest temperature measured from 1958 to 2024 between NEG and SEG (Fig. 1A), as denoted by the 95th percentile size of dots representing more variation (Suppl. File. 1). Tasiilaq (SEG) showed the highest reported temperature (mean and 95th percentile range), and Station Nord (NEG) showed the lowest recorded temperatures (Fig. 1A). There was a higher degree of variance in the temperatures in SEG compared to NEG, with a trend towards warmer temperatures in SEG (Fig. 1B).

### Higher expression of TEs in the southeastern polar bears

**Fig. 2:**
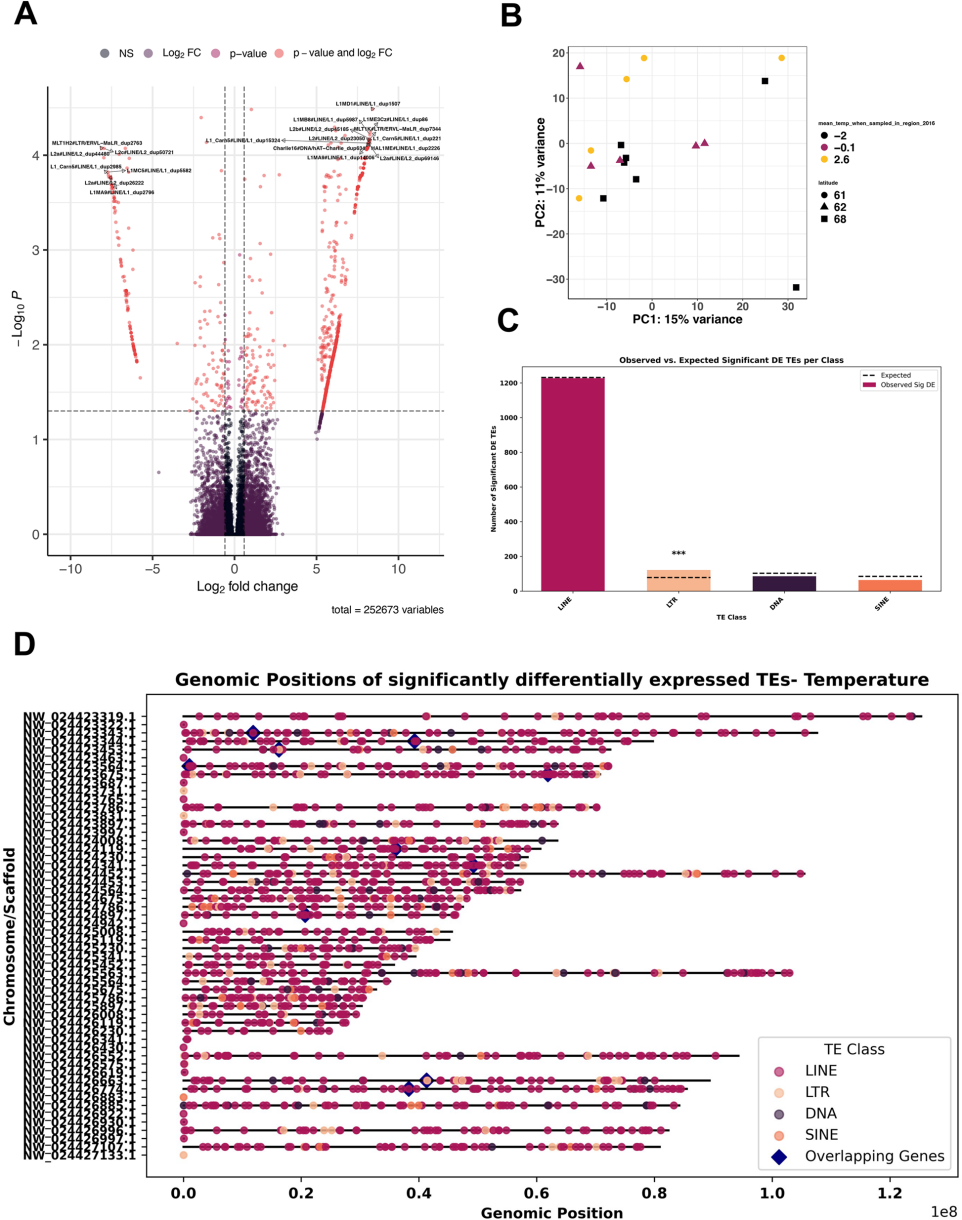
Comparison of expression and activity of TEs in NEG versus SEG bear populations profiling temperature as main effect and impact in SEG population (model: ~*sex + temperature + population*). **A** Differential expression analysis from DESeq2 analysis of TE species identified 1,500 significantly differentially expressed TEs (*p*_*adj*_ ≤ 0.05) by the Telescope pipeline, with a significance cut off at *p*_adj_ ≥ 0.05 and log_2_ fold change > 0.5 (1). **B** Principal Component Analysis of SEG and NEG polar bear populations including temperature and location. **C** Count data of significantly differentially expressed TEs at the family level as tested by hypergeometric testing (*** = *p* ≤ 0.001). **D** Genomic position of DE TE species and co-located DE genes

To further understand how temperature is associated with changes in TE expression, we designed a model implementing temperature as a covariate. This model allowed us to test whether temperature drives variation in TE expression. Differential expression analysis revealed temperature-dependent changes in TE activity (Fig. 2). Additional models including sex and population were used to assess SEG-related effects on TE expression (Suppl. Fig. 2).

When adding temperature as a covariate into our analyses, samples clustered based on temperature (Fig. 2B), and we identified 1,500 significantly DE TEs (Fig. 2A, C). The most significantly enriched TEs in the SEG population was L1MD LINE L1_dup1507, and the most significantly downregulated TE was L2a LINE/L2 dup_44480 (Fig. 2A). The most abundant TE class were again LINEs (Fig. 2C). To find genomic regions where TE activity may originate from, the genomic positions of significantly DE TE species were plotted (Fig. 2D), revealing some clustering of DE TEs on chromosomes NW_024423453.1, NW_024424119.1 and NW_02442563.1. No significantly DE TE species were identified on the Y chromosome (NW_024423336.1), but a few LINE TE species were located on the X chromosome (NW_024423319.1).

**Fig. 3:**
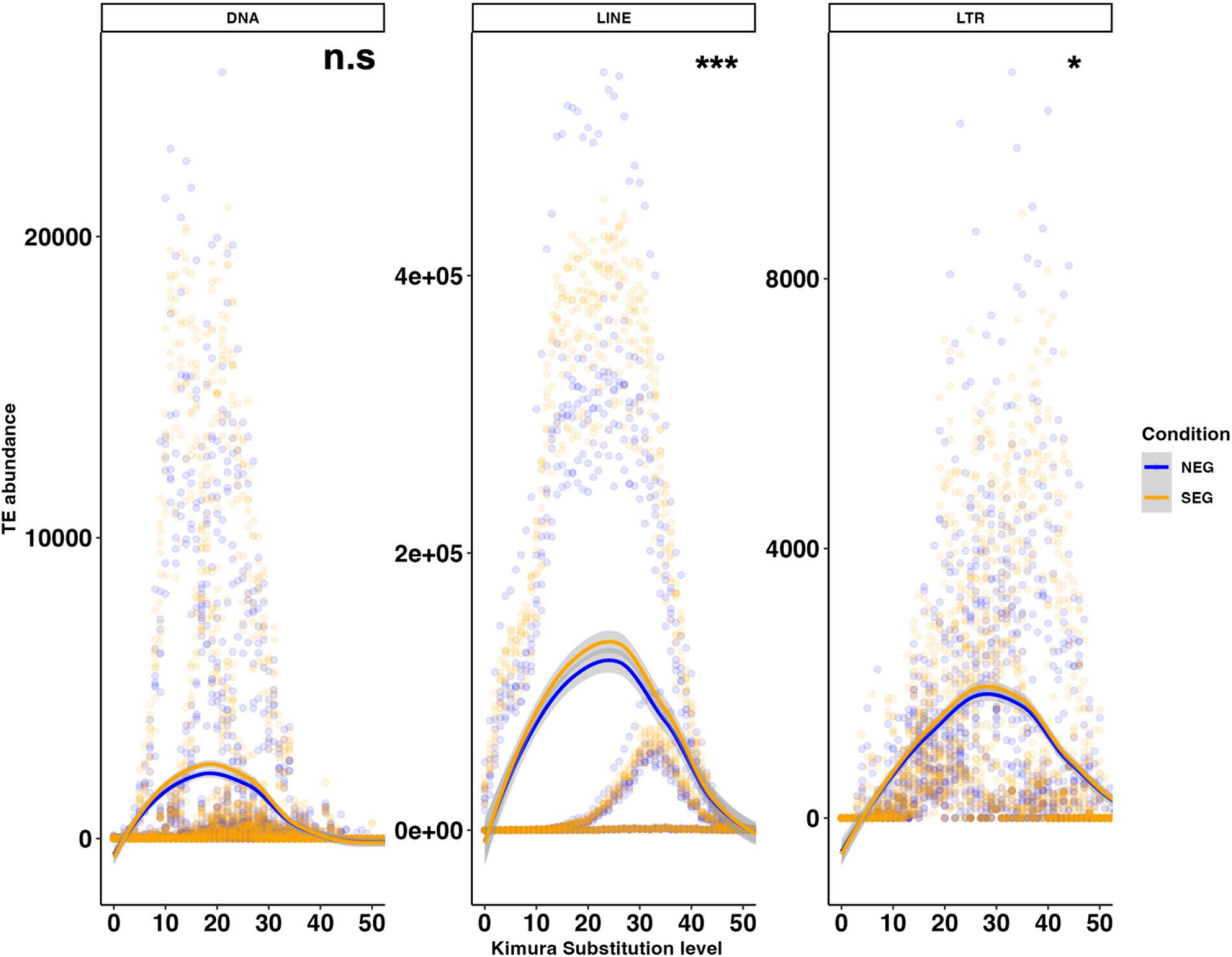
Repeat landscape of TE age and activity in NEG v SEG populations. TE age and divergence estimation by Kimura substitution analysis with RepeatMasker. The estimated divergence from the reference genome was greater for SEG bears than for NEG bears. Analysis of negative binomial testing with general linear mixed modelling results to assess the model Count ~ Condition * Div + (1|Sample), for a shift in TE age with negative binomial error distribution showed significant increase in younger TEs in SEG populations at *p* ≤ 0.05 for all LINE and LTR TEs. See Suppl. Table 1 for full statistical results. Curves show smoothened trend line of scattered points in blue for NEG and orange for SEG samples

Age estimations of DE TEs showed significant divergence in TEs between the SEG and NEG bear populations (Fig. 3), with LINEs showing the highest level of divergence, followed by DNA and LTR TEs. In the SEG population, we observed an overall increase in TE divergence and a notable peak of younger divergent LINEs (Fig. 3). To test if TE expression is enriched due to their localisation within a gene or associated promoter region, we mapped the loci of the significantly DE TEs loci against the reference genome and tested for overlap with genic regions (Suppl. Fig. 1A). We observed significant enrichment of molecular function GO terms associated to these overlapping genes (Suppl. Fig. 1). We found a significant enrichment of TEs in genic regions across the entire coding genome (Suppl. Fig. 1A). GO term analyses of TE-overlapping genes enriched in the SEG population highlighted several significantly enriched molecular functions including: hydroperoxyicosatetraenoate dehydratase activity, flippase activity and ion channel activity (Suppl. Fig. 1B).

We identified 179 significantly DE TEs (*p*_*adj*_ ≤ 0.05) between SEG and NEG bears when including just sex and population into the model (Suppl. Fig. 2B). The PCA showed modest clustering of samples based on population (Suppl. Fig. 2A). Most of the significantly DE TE species belongs to the LINE family (Suppl. Fig. 2B-C). The most significantly downregulated TE in this population was DNA TE hAT-Charlie_dup1276 from the Charlie 8 subfamily. The most significantly enriched TE was LINE TE L1_dup3552 from the L1 Carn1 subfamily (Suppl. Fig. 2B).

### Warmer temperatures linked to changes in gene expression

**Fig. 4:**
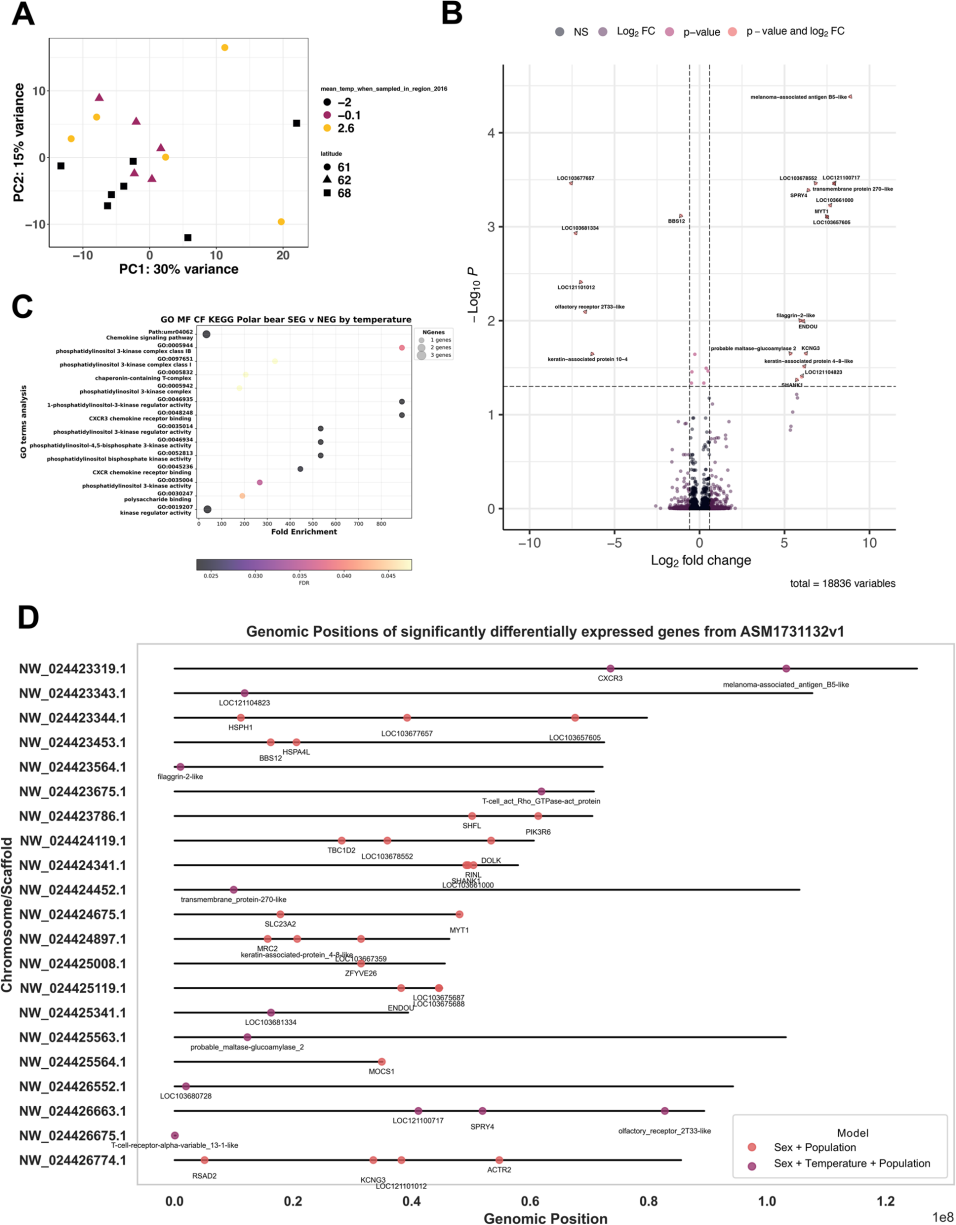
Analysis of differential gene expression to assess the main impact of temperature on the genomes in DESeq2 outputs looking at changes in SEG population. **A** Principal Component Analysis of differentially expressed genes in NEG and SEG polar bears taking temperature and sex into account shows clustering according to geographical location. **B** Volcano plot of differentially expressed genes, with a significance cut off at *p*_adj_ ≥ 0.05 and log_2_ fold change > 0.5 (1), showing 27 significantly DE genes. **C** GO terms analysis of significantly differentially expressed genes between SEG and NEG bears. **D** Loci of significantly differentially expressed genes with and without temperature covariate in *DESeq2* analyses

To further explore temperature effects on gene expression, we included temperature as a covariate in our model. This analysis identified genes whose expression varied significantly with temperature (Fig. 4). We also profiled models incorporating sex and population to evaluate SEG-related impacts on gene expression (Suppl. Fig. 3).

PCA revealed clustering of samples on PC1 with up to 30% variance explained by temperature and sampling latitude associated with local temperature and 15% in expression (Fig. 4A). We identified 27 significantly DE genes, with LOC103677657 most downregulated and LOC103668192 most enriched in the SEG population (Fig. 4B). We found no overlap in position of significantly DE genes and TEs when using sex + population covariates, but addition of temperature covariate for ASM1731132v1 revealed 10 overlapping genes with TEs (Fig. 4D). These genes were: LOC121104823, LOC103667593, LOC103678552, LOC103677657, LOC121100717, LOC121101012, BBS12 (ENSUMAG00000004608- Bardet-Biedl syndrome 12), LOC121104992, SHANK1 and LOC103660844.

Differential gene expression analysis for the model with *sex and population* as fixed variables revealed 13 significantly DE genes (Suppl. Fig. 3B). RSAD2 was most significantly downregulated, and LOC10367587 was most significantly enriched while HSPH1 and HSPA4L were significantly enriched in SEG bears (Suppl. Fig. 3B). GO term analyses of biological processes, molecular function, and cellular function for the significantly DE genes overlapping with DE TEs showed enrichment for the following terms: internal cellular dynamics and movements: chromosome movement towards spindle pole, PRC1 complex, organic acid: sodium symporter activity, negative regulation of protein localization to nucleus, and immune responses: negative regulation of viral genome replication, defence response to virus, defence response to symbiont, (Suppl. Fig. 3C).

Analysis of the raw RNA-seq reads mapped to 10-kb bins across the whole genome showed some significant clustering of the samples independently of any annotation and alignment (Suppl. Fig. 4). Despite this the above results show some statistical links to temperature elevation in the SEG bear population that are potentially affecting their genome structure.

## Discussion

SEG bears in warmer Greenland towns exhibited significantly DE TEs and genes compared to NEG bears (Figs. 1-2). LINEs were the most abundant DE TEs and LTR TEs were significantly enriched in SEG bears. Many DE TEs overlapped with genic regions, and Kimura analyses revealed more potentially recent activity by DNA, LINE, and LTR TEs in SEG bears (Figs. 2, 3 and 4). Additionally, heat-shock protein genes HSPA4L and HSPH1 were significantly enriched in SEG bears (Suppl. Fig. 3B). Taken together, our results suggest that TE activity in SEG bears is increased and may contribute to the differential expression of genes between the two populations. Below, we discuss the implications of our findings in a more general context and how they may help understanding adaptation in wild animal populations more generally.

###  Meteorological analysis

Polar bears are dependent on sea ice, and the warmer and more variable SEG climate (Fig. 1A), is forecast to be the norm for all polar bears by the end of the 21 st century [14]. The warmest temperatures in this study were observed in Tasiilaq (Fig. 1B), ranging between − 30 °C and + 26 °C, which is characterised by a deep fjord and mountainous habitat with some drifting sea pack ice as evidence of how temperature is isolating the SEG bears [6, 40].

### Genetic divergence between SEG and NEG polar bears

A previous study reported SEG bears are more genetically diverged from NEG bears than other polar bear populations in Greenland [14]. An *F*_ST_ (fixation index) analysis is a quantified measure of genetic variation between versus within populations [41]. This conclusion was supported by their *F*_ST_ analysis to measure genetic differentiation between populations, with a score of 0 meaning no differentiation and a 1 score meaning complete differentiation, a score of 0.05–0.15 is showing moderate differentiation, 0.15–0.25 high differentiation. Their study observed an *F*_ST_ = 0.059, indicative of moderate differentiation between the SEG and NEG populations of bears. In line with what we observed in our differential TE and gene expression analysis, where we see significant differences in the SEG transcriptomes (Figs. 2, 3 and 4).

The genetic isolation of the SEG bears is likely due to the limited gene flow caused by the East Greenland coastal current [14], restricting bear movements and thus promoting genetic drift over several hundred years of isolation. Despite this divergence, a previous study using the same dataset reported no significantly DE genes, in contrast to our findings of 27 DE genes. This discrepancy may be attributed to differences in model design, such as the inclusion of intercept terms and the specific covariates considered during analysis. The previous analyses were based on data from 16 adult samples, whereas we used 17 adult samples and used more stringent *DESeq2* analysis, filtering out all genes with less than 10 reads, compared to the 1 read threshold previously analysed. The PCA showed similar clustering patterns between our study and the previous study.

### Differential TE expression analysis

The main clusters of DE TEs across all samples identified in our analyses are LINEs, and they were also the most abundant and significantly enriched in SEG bears (Fig. 2). LINEs and SINEs are the most abundant family of TEs in mammalian genomes in general, with limited LTR and DNA TE retention [11]. The transcripts from DNA TEs detected in our analysis are likely the result of read-through from genes, which is unsurprising given that many TEs in this study overlap with genic and coding regions of the genome (Suppl. Fig. 1A). LINEs and SINEs are also the most mobile and active TEs in bear genomes, and are most active in brown bears, followed by polar bears [12].

Our results showed the impact of temperature on gene expression, which also affected the clustering of the samples (Fig. 2B). This association of temperature with genome mobility in polar bear aligns with similar studies in model systems including *Arabidopsis* [42], *C. elegans* [43] and *Drosophila* [44], where environmental stress was reported to increase TE mobility. Future work could include the use of long-read DNA sequencing and small RNA sequencing data to quantify TE insertions and differential expression analysis of piRNAs and to link our expression analysis with TE mobility at the DNA level.

Our finding of significantly DE TEs is in line with our idea of a putative role for TEs in the adaptation of SEG polar bears and offers new avenues to understanding rapid adaptation to changing environmental conditions. Divergent TEs between populations have also been described in great pandas, where LINE and SINE TEs were generally younger in age, indicative of recent mobilisation events [13]. We found significantly elevated expression of LTR TEs in the SEG population (Fig. 2C). LINE have been previously shown to be important in the evolution of regulatory elements in the mammalian genome [45]. In fact, we observed a peak of younger, diverged LINEs in the SEG population of polar bears compared to the reference TEs present in the genome (Fig. 3). Older TEs included a modest amount of DNA and LTR TEs in older genome expansion events, although these divergent events constitute a smaller fraction of the genome. LINE TEs represent 38% of the polar bear genome, and 54% of the Y-chromosome scaffolds [46]. Interestingly, in the Giant panda, a full-length LINE TE related to the L1-1_AMe TE was thought to have been recently active [46]. Future long-read DNA sequencing will further allow us to study the integration of TEs into the genome and allow the identification of putative functional copies of TEs. Overall, the observed significant enrichment of expressed young TEs in SEG bears could be in response to their challenging habitat and climate, but we need further evidence to fully appreciate their integration back into the genome.

Gene Ontology (GO) term enrichment analysis revealed several biological processes that were significantly enriched for the genes overlapping with DE TEs. These terms highlighted roles in a number of biological processes and one of the key pathways, hydroperoxyicosatetraenoate dehydratase activity, is linked to oxidative stress and lipid peroxidation processes, which are also involved in thermal and subsequent nutritional stress in polar bear populations [47, 48]. This is interesting as warmer environments can affect polar bear diet, and if polar bears have lower fat reserves they are more likely to have failed pregnancies, with data previously showing a 28% reduction in the number of pregnant female bears due to warmer environments [49]. This could also lead to hormonal imbalances caused by temperature rise and nutritional deficits in the SEG population [50]. Molecular functions for flippases were also significantly enriched, which are integral for maintenance of membrane integrity and signalling especially under thermal fluctuations [51]. Other enriched terms included gated channel and chloride channel activity, which may be involved in adaptation to thermal challenges and improve thermoregulation via efficient ion movement and osmotic balance, as reported in Antarctic fish species group, cryonotothenioids [52]. As suggested previously, there is a great need for the development of a rapid test that measures polar bear health in response to environmental stress [47], and our data may be useful towards genomic testing of environmentally induced changes in TE activity and gene expression for profiling of both recent and past environmental stress events.

Increased TE activity has been shown to lead to immune responses in the host due to detection of DNA damage and subsequent inflammatory responses [53]. This may explain the enrichment of processes for immune signalling pathways associated with significantly DE genes (Fig. 4B-C), and the genes that overlap with loci of significantly DE TEs (Suppl. Fig. 1). We estimate that the recent spike in TE activity, as demonstrated by the significant peak of younger TEs (Fig. 3) could be representative of at least a few hundred years, this would be enough to cover several generations of polar bears and is of sufficient time to allow for introduction of new genetic variability due to TE activity to favour rapid adaptation [8, 54].

While our study is assessing TE activity in a somatic tissue (blood), adaptation would rely on germline TE activity. Some TEs are tissue specific, and their activity is generally less tightly regulated in somatic tissues [55], providing a direct profile of a response to environmental stress and possible resulting genetic divergence that may in time lead to genomic adaptation [8]. Nevertheless, the finding of differential TEs in a somatic tissue and the age difference in TEs between the two populations, suggests an overall difference in TE activity across several generations of bears in these two populations, possibly in response to differential environmental factors.

### Differential gene expression analysis

The evolutionary origins of bears as a taxonomic group are complicated, and they are thought to have evolved over the past 5 million years in harsh and rapidly changing environments [56]. To better understand polar bear genomes, we therefore profiled the differential gene expression of SEG versus NEG polar bears, whilst using *sex* as a covariate to account for differences in sample numbers and sex biases in gene expression. Given the differences in habitat over the recent decades of extreme warming and variable weather patterns in SEG [6], it is unsurprising that SEG bears are starting to show significant differences in gene expression compared to NEG bears.

### Linking gene and TE profiles in response to temperature

Our re-analyses of gene expression data using more conservative filtering steps revealed a number of significantly DE genes between the two populations when taking sex and location into account, which contrasts with earlier results on the same dataset reporting no significant DE genes [14]. Upregulated DE genes in SEG bears included RSAD2 (downregulated), ACTR2, HSPA4L and HSPH1 (enriched) (Suppl. Fig. 3B), where RSAD2 (also known as Viperin) is an evolutionarily conserved antiviral protein-coding gene [57]. ACTR2 (actin-related protein 2) in house mice *Mus musculus* is involved in thrombosis, and mutations in the gene have been found to be linked to thrombo-suppression [58] whereas HSPA4L (Hsp70) and HSPH1 (Hsp110) are highly conserved and related families of proteins known as heat-shock proteins universal to all eukaryotic species so far, acting as molecular chaperones [59]. This is interesting as other Hsp chaperones, including *Hsp90*, regulate protein folding, unfolding and degradation [60]. *Hsp90* and *Hsp70* are also important in piRNA biogenesis and silencing of TEs, and function in elevated temperature environments [61]. The most significant differential expression in TEs was observed in the analysis with the addition of temperature as a covariate (Fig. 2A). This is supported by evidence in *Drosophila* where TEs are activated in response to heat shock due to *Hsp70* and other chaperones having a role in the collapse of piRNA production, and thus further TE action [62]. Combined, this is evidence that the host genome is potentially responding to the changes in environmental temperature.

When taking temperature as covariate into account (Fig. 4B), out of our 27 significantly DE genes, 10 of these gene loci overlapped with significantly DE TEs, these included: LOC121104823, LOC103678552, LOC121100717, and LOC121104992 (all enriched), LOC121101012 (downregulated) are all long non-coding RNAs (lncRNAs) (Fig. 4B, D). Long non-coding RNAs are highly abundant in mammals, with over 80% of transcribed products generating lncRNAs that have roles in chromatin organization and gene regulation [63]. The accessibility of chromatin, and methylation is significant as this can lead to de-repression of TEs [64].

The most significantly downregulated gene in the SEG population was LOC103677657, a pseudogene with unknown function. BBS12, an mRNA for Bardet-Biedl syndrome 12 protein, involved in chaperone protein complex assemblies, and orthologous with human *BBS12*, was also downregulated in SEG bears. Mutations or loss of BBS12 in humans lead to cilia abnormalities [65]. Significantly enriched genes in SEG bears with reported functions included LOC103667593, which is a predicted mRNA for filaggrin-2-like protein linked to inflammatory skin diseases in human [66], and LOC121104992, a predicted mRNA for keratin-associated 4–8-like protein. SHANK1 was also significantly upregulated in SEG bears and is an mRNA for SH3 and multiple ankyrin repeat domains protein 1 and orthologous to human *SHANK1*. *SHANK1* encodes a scaffold protein and can inhibit the activation of integrins [67], which can lead to changes in cell signalling. This links to the GO term processes observed for phosphatidylinositol activity highlighting cellular activity and signalling (Fig. 4C). Other GO terms in response to the warmer SEG climate showed enrichment for terms related to immune responses including defence response to virus, and negative regulation of viral genome replication (Suppl. Fig. 3C). TE invasion is known to induce an immune response in mammals, activating the interferon and innate immune pathways [68]. Polar bears in regions of reduced sea ice habitat are at higher risk of pathogen exposure [69], which may explain the changes in SEG bears. However, whether SEG bears are indeed exposed to higher pathogen loads needs further careful investigations.

High-quality reference genomes and long-read sequence data are beneficial for understanding evolutionary processes in the genome [70]. Despite this most tools that are available for the analysis of TE activity have been developed for use with short-read data [71], likely due to budget and sample availability. Benefits of long-read sequence data include improved resolution and accuracy in repetitive regions [71]. The ability to capture a full-length region, such as a TE, and the surrounding genomic features would support the analysis of structural variation, with recent work showing nearly half of TE insertions were missed in short read data compared to long-read data [70]. More tools are becoming available for this type of analysis [72], and future work should look to access samples where possible, to generate some data for both Ursus maritimus and other endangered species.

## Conclusions

Our analysis indicates notable differences between the transcriptomes of SEG versus NEG bears, particularly in TE expression, which may be influenced by the more variable and higher temperatures experienced by SEG bears. Our study provides a basis for further work into TEs in a wider subset of polar bear subpopulations and highlights potential candidate TE species for further investigation as potential markers of environmental stress. Future work to revise and further annotate the *Ursus maritimus* genome, at all levels not just TEs will prove highly valuable in the efforts to preserve this vulnerable species through genomic analyses. Larger scale analyses across all other subpopulations of polar bear, and comparative analyses across different bear species in a range of climate zones may prove highly valuable for the planning of conservation efforts and species management.

## Supplementary Information

Supplementary Material 1: Supplementary Figure 1- Significantly DE TE loci and their overlap with genomic features and genes in NEG v SEG populations. (A) Abundance of TEs overlapping genomic features, statistical enrichment analysed if this overlap was more significant than to be expected by chance (χ ^2^ test of independence), *p* ≤ 0.001, as denoted by ***. SINEs were most significantly enriched in transcript regions (χ² = 2954.60 ) and LINEs were most significantly depleted in transcript regions (χ² = 306.67). Stars in red show significant enrichment, stars in blue denote significant depletion. (B) GO terms of molecular functions of genes overlapping with significantly enriched TEs for molecular functions from *ShinyGO* v0.77. Supplementary Figure 2- Comparison of expression and activity of TEs between SEG and NEG bears with sex + population as fixed factors. (A) PCA analysis of NEG v SEG bears show clustering based on geographical location of sample for TE expression. (B) Differential expression in DESeq2 analysis of TE species identified 179 significantly differentially expressed TE copies (p_adj_≤ 0.05 ). (C) Count data of significantly differentially expressed TEs at the family level. Supplementary Figure 3*** –*** Differential expression analysis in DESeq2 using RNA-sequencing data from samples in PRJNA669153 aligned to the reference genome ASM1731132v1 to compare NEG and SEG bears and observe impact of sex+ population . (A) Principal Component Analysis shows variation in gene expression correlates to geographical location of the sample. (B) Volcano plot of the differentially expressed genes observed following DESeq2 analysis, with 13 significantly differentially expressed genes in total, with a significance cut off at p_adj_ ≥ 0.05 and log_2_ fold change >0.5 (1). (C) GO terms analysis, examining biological processes (BP), Molecular function (MF) and cellular function (CF). All genes expressed in the study were used as a background list, and those with *p* ≤ 0.1 processed for GO terms analysis with ShinyGO v0.77. Supplementary Figure 4 – Analysis of sample clustering on raw RNA-seq reads. PCA analysis with all RNA-seq reads, mapped to 10-kb bins across the whole genome generated with MultiBamSummary in Deeptools package. Significant clusters are indicated by the coloured ellipses for NEG (blue) and SEG (orange). Supplementary Table 1 – Statistical analysis of models to examine a shift in TE family age from Kimura Substitution analysis data from RepeatMasker outputs. General linear modelling results to assay the model Count ~ Condition * Div + (1|Sample), based on Akaike Information Criterion (AIC) and overdispersion values. Lower AIC values indicate better model fit. The Negative Binomial model is preferred over the Poisson model due to significant overdispersion in TE counts. The lower section of the table presents the results of a shift analysis, showing the estimated shift change, statistical significance (*p*-value), and the inferred shift direction in SEG polar bears negative shift values and significant indicate younger TEs that are enriched in SEG samples. Supplementary files.

## Data Availability

RNA-seq data was deposited by Laidre et al. 14 under ENA accession PRJNA669153. All R and python scripts used to analyse this data can be found in the following Github repository: https://github.com/alicegodden/polarbear/. Metadata used for the files accessed can be found in Supplementary file 2. The datasets supporting this article have been uploaded as part of the supplementary material in files 1-8. Supplementary files include all raw counts data from RNA-seq analyses, DESeq2 outputs for differential gene and TE expression with different models and can be found here: https://github.com/alicegodden/polarbear/tree/main/supplementary_data. Full length Supplementary File 6, and other input files used in the bioinformatic pipelines also provided at Zenodo here: https://doi.org/10.5281/zenodo.17573136 (https://zenodo.org/records/17573136). We also provide Suppl. File 9 which includes metadata for samples analysed in the RNA-seq analysis, and Suppl. File 10 with the configuration settings for our RNA-seq on our local cluster for full replication.

## Abbreviations

DE: Differentially expressed
DMI: Danish Meteorological Institute
DNA-seq: DNA sequencing
lncRNAs: Long non–coding RNAs
NEG North: East Greenland
piRNA: PIWI–interacting RNA
RNA-seq: RNA sequencing
SEG: South–East Greenland
TEs: Transposable elements
