# Supplementary material for: Diverging transposon activity among polar bear sub-populations inhabiting different climate zones
Source: Mob DNA. 2025 Dec 12;16:47. doi: 10.1186/s13100-025-00387-4 (PMC12699835; doi:10.1186/s13100-025-00387-4)
Supplement: Supplementary file 1 — Supplementary Material 1: Supplementary Figure 1- Significantly DE TE loci and their overlap with genomic features and genes in NEG v SEG populations. (A) Abundance of TEs overlapping genomic features, statistical enrichment analysed if this overlap was more significant than to be expected by chance (χ 2 test of independence), p ≤ 0.001, as denoted by ***. SINEs were most significantly enriched in transcript regions (χ² = 2954.60 ) and LINEs were most significantly depleted in transcript regions (χ² = 306.67). Stars in red show significant enrichment, stars in blue denote significant depletion. (B) GO terms of molecular functions of genes overlapping with significantly enriched TEs for molecular functions from ShinyGO v0.77. Supplementary Figure 2- Comparison of expression and activity of TEs between SEG and NEG bears with sex + population as fixed factors. (A) PCA analysis of NEG v SEG bears show clustering based on geographical location of sample for TE expression. (B) Differential expression in DESeq2 analysis of TE species identified 179 significantly differentially expressed TE copies (padj≤ 0.05 ). (C) Count data of significantly differentially expressed TEs at the family level. Supplementary Figure 3 – Differential expression analysis in DESeq2 using RNA-sequencing data from samples in PRJNA669153 aligned to the reference genome ASM1731132v1 to compare NEG and SEG bears and observe impact of sex+ population . (A) Principal Component Analysis shows variation in gene expression correlates to geographical location of the sample. (B) Volcano plot of the differentially expressed genes observed following DESeq2 analysis, with 13 significantly differentially expressed genes in total, with a significance cut off at padj ≥ 0.05 and log2 fold change >0.5 (1). (C) GO terms analysis, examining biological processes (BP), Molecular function (MF) and cellular function (CF). All genes expressed in the study were used as a background list, and those with p ≤ 0.1 processed for [file 13100_2025_387_MOESM1_ESM.docx]

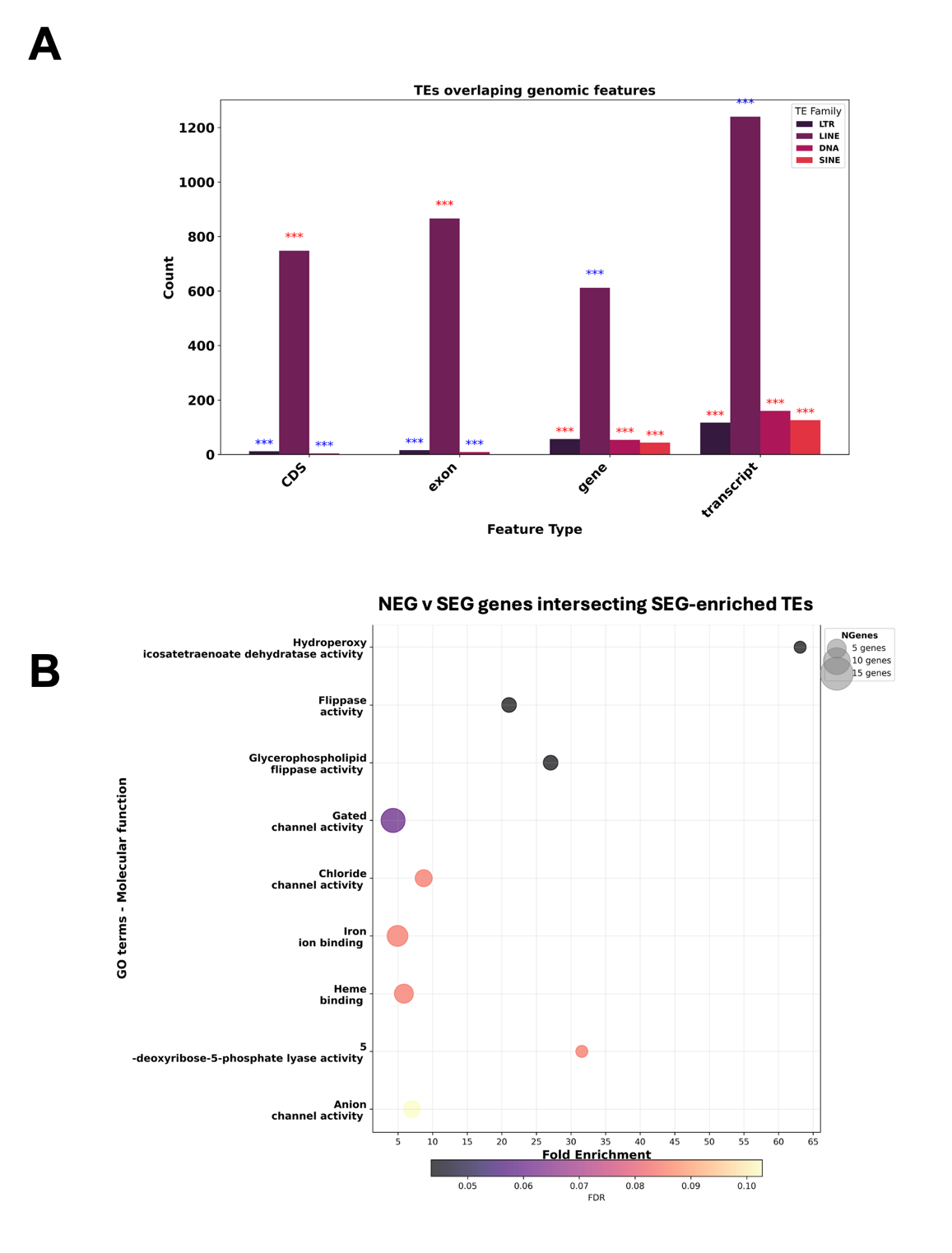


**Supplementary Figure 1**- Significantly DE TE loci and their overlap with genomic features and genes in NEG v SEG populations. **(A)** Abundance of TEs overlapping genomic features, statistical enrichment analysed if this overlap was more significant than to be expected by chance (χ ^2^ test of independence), *p* ≤ 0.001, as denoted by ***. SINEs were most significantly enriched in transcript regions (χ² = 2954.60 ) and LINEs were most significantly depleted in transcript regions (χ² = 306.67). Stars in red show significant enrichment, stars in blue denote significant depletion. **(B)** GO terms of molecular functions of genes overlapping with significantly enriched TEs for molecular functions from *ShinyGO* v0.77


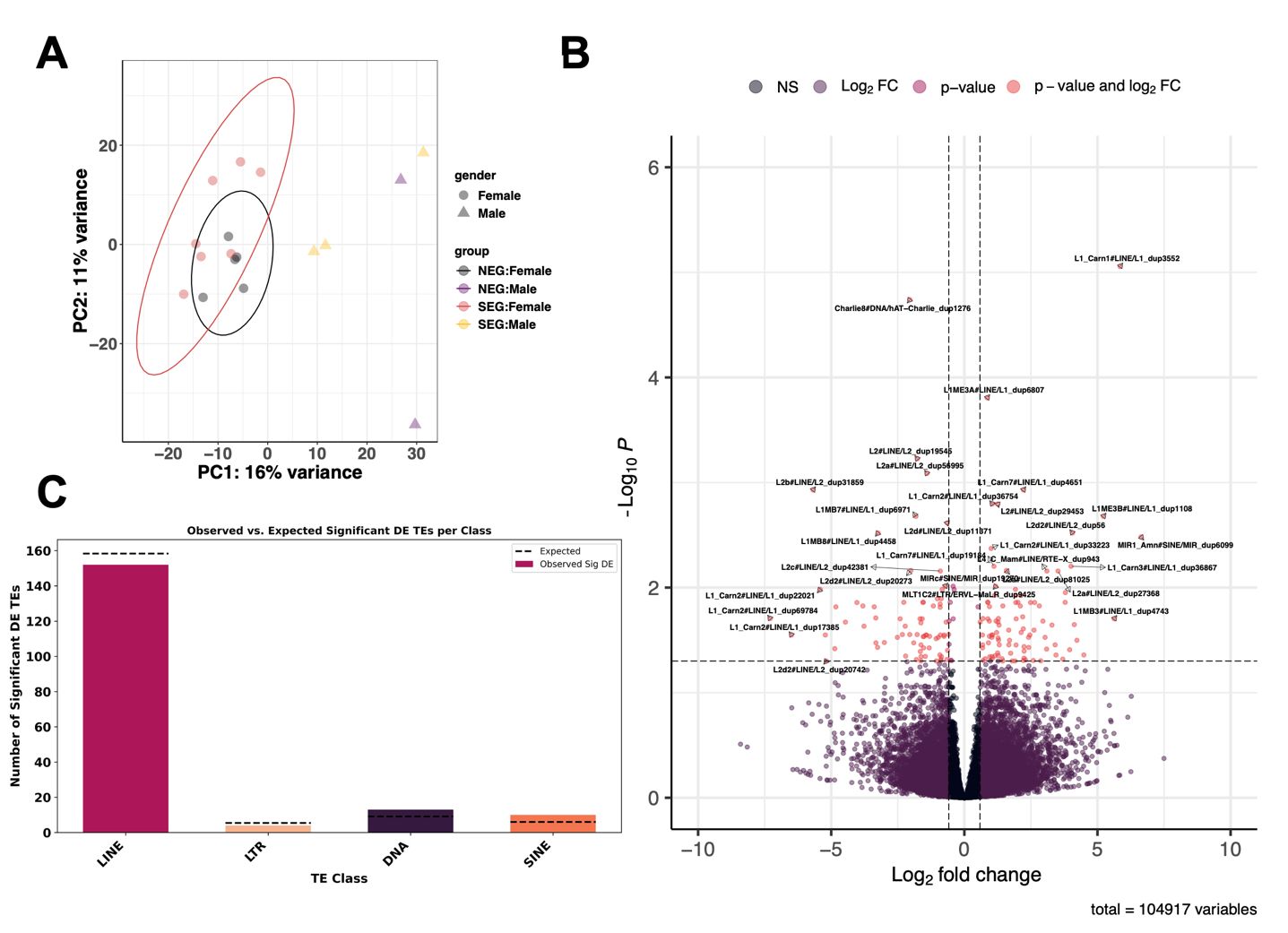


**Supplementary Figure 2**- Comparison of expression and activity of TEs between SEG and NEG bears with sex + population as fixed factors. **(A)** PCA analysis of NEG v SEG bears show clustering based on geographical location of sample for TE expression. **(B)** Differential expression in DESeq2 analysis of TE species identified 179 significantly differentially expressed TE copies (p_adj_ ≤ 0.05 ). **(C)** Count data of significantly differentially expressed TEs at the family level.


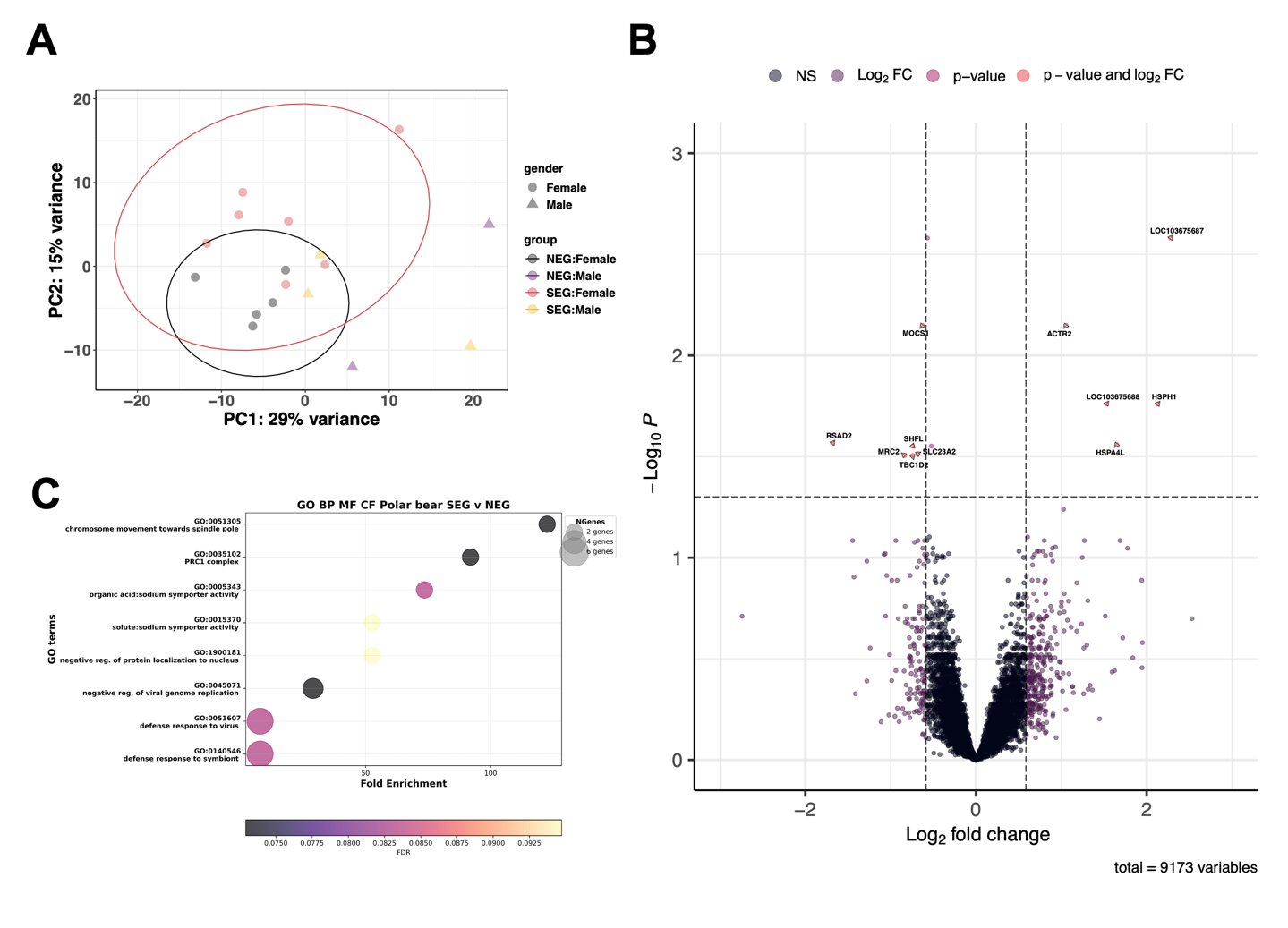


**Supplementary Figure 3 –** Differential expression analysis in DESeq2 using RNA-sequencing data from samples in PRJNA669153 aligned to the reference genome ASM1731132v1 to compare NEG and SEG bears and observe impact of sex + population . **(A)** Principal Component Analysis shows variation in gene expression correlates to geographical location of the sample. **(B)** Volcano plot of the differentially expressed genes observed following DESeq2 analysis, with 13 significantly differentially expressed genes in total, with a significance cut off at p_adj_ ≥ 0.05 and log_2_ fold change > 0.5 (1). **(C)** GO terms analysis, examining biological processes (BP), Molecular function (MF) and cellular function (CF). All genes expressed in the study were used as a background list, and those with p ≤ 0.1 processed for GO terms analysis with ShinyGO v0.77.

**Supplementary Figure 4 –** Analysis of sample clustering on raw RNA-seq reads. PCA analysis with all RNA-seq reads, mapped to 10-kb bins across the whole genome generated with MultiBamSummary in Deeptools package. Significant clusters are indicated by the coloured ellipses for NEG (blue) and SEG (orange).

**Supplementary Table 1 –** Statistical analysis of models to examine a shift in TE family age from Kimura Substitution analysis data from RepeatMasker outputs. General linear modelling results to assay the model Count ~ Condition * Div + (1|Sample), based on Akaike Information Criterion (AIC) and overdispersion values. Lower AIC values indicate better model fit. The Negative Binomial model is preferred over the Poisson model due to significant overdispersion in TE counts. The lower section of the table presents the results of a shift analysis, showing the estimated shift change, statistical significance (p-value), and the inferred shift direction in SEG polar bears Negative shift values and significant indicate younger TEs that are enriched in SEG samples.

| ***TE_Family*** | ***Model_Type*** | ***AIC*** | | ***Overdispersion*** |
| --- | --- | --- | --- | --- |
| *DNA* | Poisson | Not fitted | | NA |
| *DNA* | Negative Binomial | Not fitted | | NA |
| *LINE* | Poisson | 482042639 | | 178219.252 |
| *LINE* | Negative Binomial | 433921730 | | NA |
| *LTR* | Poisson | 8657218.76 | | 2508.73126 |
| *LTR* | Negative Binomial | 8373281.52 | | NA |
| ***TE_Family*** | ***Model_Type*** | ***Div_estimate*** | ***P value*** | ***Shift direction in SEG*** |
| *DNA* | Not fitted | NA | *n.s* | none |
| *LINE* | Negative binomial | -0.0324049 | *<0.001/**** | Younger |
| *LTR* | Negative binomial | -0.014434 | *0.0310237/** | Younger |

**SUPPLEMENTARY FILES**

Supplementary files can be found here: <https://github.com/alicegodden/polarbear/tree/main/supplementary_data>

With Supplementary file 4, and other input files used in the bioinformatic pipelines also provided at Zenodo here: https://doi.org/10.5281/zenodo.17573136 (<https://zenodo.org/records/17573136>)

**Supplementary File 1-** Meteorological data accessed from DMI **–** “Suppl. File. 1- Temperature_data - DMI.csv”

**Supplementary File 2**- Metadata of samples used for the RNA-seq analysis “Suppl. File. 2. bear_metadata_adult.csv”

**Supplementary File 3 –** Telescope raw counts RNA-seq- TEs ASM1731132v1: “Suppl. File 3-Telescope raw counts RNA-seq- TEs ASM1731132v1.zip” compressed csv file.

**Supplementary File 4 –** Telescope DESeq2 data RNA-seq ASM1731132v1: “Suppl. File 4- Telescope DESeq2 data RNA-seq ASM1731132v1.csv” <https://zenodo.org/records/17573136>

**Supplementary File 5-** DESeq2 data RNA-seq ASM1731132v1 Temperature effect on TE expression: “Supp. File. 5- Temperature_effect_results_newgen_bear_TelescopeTEs.txt”

**Supplementary File 6 –** Raw counts RNA-seq ASM1731132v1: “Suppl. File 6-Raw counts RNA-seq ASM1731132v1.tsv”

**Supplementary File 7 –** DESeq2 data RNA-seq ASM1731132v1: “Suppl. File 7-DESeq2 data RNA-seq ASM1731132v1.csv”

**Supplementary File 8-** DESeq2 data RNA-seq ASM1731132v1 Temperature effect: “Suppl. File. 8- Temperature_effect_results_newgen_bear.csv”

**Supplementary File 9**- Metadata of samples used for the RNA-seq analysis with temperature and latitude information “Suppl. File. 9. bear_adult_temp_metadata.csv”

**Supplementary File 10-** Configuration file used with nf-core RNA-seq pipeline “Suppl. File. 10. bear.conf”
